# An Example of Neuro-Glial Commitment and Differentiation of Muse Stem Cells Obtained from Patients with *IQSEC2*-Related Neural Disorder: A Possible New Cell-Based Disease Model

**DOI:** 10.3390/cells12070977

**Published:** 2023-03-23

**Authors:** Sura Hilal Ahmed Al Sammarraie, Domenico Aprile, Ilaria Meloni, Nicola Alessio, Francesca Mari, Marianna Manata, Caterina Lo Rizzo, Giovanni Di Bernardo, Gianfranco Peluso, Alessandra Renieri, Umberto Galderisi

**Affiliations:** 1 Department of Experimental Medicine, Luigi Vanvitelli Campania University, 80138 Naples, Italy; 2 Department of Medical Biotechnology, University of Siena, 53100 Siena, Italy; 3 Sbarro Institute for Cancer Research and Molecular Medicine, Center for Biotechnology, Temple University, Philadelphia, PA 19122, USA; 4 UniCamillus, 00131 Rome, Italy; 5 Genome and Stem Cell Center (GENKÖK), Erciyes University, 38280 Kayseri, Turkey

**Keywords:** intellectual disability, disease model, adult stem cells

## Abstract

Although adult stem cells may be useful for studying tissue-specific diseases, they cannot be used as a general model for investigating human illnesses given their limited differentiation potential. Multilineage-differentiating stress-enduring (Muse) stem cells, a SSEA3(+) cell population isolated from mesenchymal stromal cells, fat, and skin fibroblasts, may be able to overcome that restriction. The Muse cells present in fibroblast cultures obtained from biopsies of patients’ skin may be differentiated into cells of interest for analyzing diseases. We isolated Muse stem cells from patients with an intellectual disability (ID) and mutations in the IQSEC2 gene (i.e., BRAG1 gene) and induced in vitro neuroglial differentiation to study cell commitment and the differentiation of neural lineages. The neuroglial differentiation of Muse cells revealed that IQSEC2 mutations may alter the self-renewal and lineage specification of stem cells. We observed a decrease in the percentage of SOX2 (+) neural stem cells and neural progenitors (i.e., SOX2+ and NESTIN+) in cultures obtained from Muse cells with the mutated IQSEC2 gene. The alteration in the number of stem cells and progenitors produced a bias toward the astrocytes’ differentiation. Our research demonstrates that Muse stem cells may represent a new cell-based disease model.

## 1. Introduction

The study of the biomolecular phenomena underlying human diseases is paramount to developing strategies for their treatment and/or prevention. To date, several animal models have been used as tools to clarify disease-related molecular mechanisms, and the use of transgene transfer and other genetic techniques has made such models critical in medical investigations. Nevertheless, during evolution, animals, including mammals, have accrued many developmental, genetic, biomolecular, and physiological differences whose occurrence may render some research for disease treatments useless [1,2]. After all, experiments performed on animals may not translate into replications in human clinical trials [3].

To overcome such hurdles, human-cell-based disease models have been developed in recent decades. Models for human pathologies may be generated by using pluripotent stem cells (PSCs), which are normal primary cell lines with nearly unlimited capacity for self-renewal and the potential to adopt any cellular phenotype via differentiation. Somatic cells, primarily fibroblasts, from patients may be reprogrammed using defined factors to produce induced PSCs (iPSCs) [2]. The use of iPSCs has shown outstanding results in biomedicine and drug discovery, especially for neurological and cardiological diseases, given the difficulty of isolating and cultivating neural and cardiac cells from patients for specific medical approaches. The fibroblasts obtained from a patient’s biopsy may be cultivated in vitro and reprogrammed into iPSCs, which can be differentiated into disease-relevant cell types [2,4]. However, the use of iPSCs has some limitations due to the genetic variability associated with the reprogramming process. Some research has shown that iPSCs may have residual epigenetic memory from the cells from which they were derived [5,6,7,8]. Moreover, choosing an optimized method for the cellular transfection of the reprogramming molecules used to obtain iPSCs can pose certain questions [9,10,11].

Aside from iPSCs, other adult stem cells may be useful for modeling cell-based diseases. Multilineage-differentiating stress-enduring (Muse) stem cells are a SSEA3(+) cell population isolated from the mesenchymal stromal cells residing within bone marrow and subcutaneous fat, which correspond to 2–3% of the total cell population, and from skin fibroblasts, which represent 1% of the cell population [12,13,14]. Muse stem cells express pluripotent genes, are self-renewable, and are able to differentiate into triploblastic cell phenotypes from a single cell [15]. They can locate within damaged tissue. For that reason, it has been proposed that they may support tissue repair by differentiating in the constituent cells within tissues [13,14,16]. Muse stem cells can also be isolated from a patient’s skin biopsy and induced to differentiate into cells of interest for drug discovery and analyzing diseases. We aimed to test this hypothesis by determining if Muse stem cells may represent a new cell-based disease model. In this context, we isolated Muse stem cells from patients with the *IQSEC2* gene mutation.

The *IQSEC2* gene (i.e., *BRAG1* gene) encodes for several protein isoforms that vary at the N- and C-termini and share a common region, which includes a coiled–coiled domain followed by an IQ-like domain with regulatory function, a catalytic Sec7 domain, a pleckstrin homology domain, and a PDZ-binding domain [17]. IQSEC2 proteins, meanwhile, are guanine nucleotide exchange factors for the RAS superfamily GTPase, including ARF6, which IQSEC2 isoforms activate by exchanging their GDP for GTP via the Sec7 domain. IQSEC2 proteins, albeit present in several tissues and organs, are most strongly expressed in the cerebral cortex, cerebellum, olfactory bulb, and hippocampus. In those brain structures, IQSEC2 is localized at the postsynaptic dense regions of excitatory synapses and is involved in trafficking synaptic receptors by regulating endocytosis and Golgi–endoplasmic reticulum activities [18,19].

Mutations in the coding region of *IQSEC2* have been identified in patients with intellectual disability (ID), epilepsy, and autism since 2008 [17,20]. Wide phenotypic diversity has been described for *IQSEC2*-related ID, as is also the case for Rett syndrome (RTT). Indeed, several patients with *IQSEC2* mutations show clinical symptoms similar to RTT, and some meet all of the criteria for canonical RTT [20]. Given the heterogeneity of the clinical symptoms associated with *IQSEC2* mutations, patient-specific tools that can clarify the disease-associated biological and molecular pathways may be important for therapeutic decisions in the context of personalized medicine. Against that background, we isolated Muse stem cells from patients with ID and mutations in the *IQSEC2* gene and induced in vitro neuroglial differentiation to study cell commitment and the differentiation of neural lineages.

## 2. Materials and Methods

### 2.1. Participants

Three female patients with ID (mean age = 13.06 ± 6.5 years), as well as a healthy female control patients 13 years of age, participated in the study.

A 3 mm skin punch biopsy was performed after the written informed consent of either the parents or the legal guardians of the patients was obtained. Biopsies from the patients were performed in the Dermatology Unit of the Azienda Ospedaliera Universitaria Senese (Siena, Italy), whose Institutional Review Board approved the study.

### 2.2. Fibroblast Isolation

Fibroblasts were isolated and cultured following standard published protocols [21]. To begin, cells were cultivated in 10 cm dishes with low-glucose D-MEM (MicroGEM, Napoli, Italy) supplemented with 10% ES-FBS (Euroclone, Pero, Italy). Next, we incubated the cells for 7–10 d in culture medium in order to reach confluence (i.e., P0). We later trypsinized and grew the cells until the third in vitro passage (i.e., P3).

As a control, we also used primary human normal dermal fibroblasts (code PCS-201-012) obtained from ATCC Italy and from Lonza Bioscience Italy (code CC-2511). The cells were cultivated according to the manufacturers’ instructions.

### 2.3. Culture of Muse Stem Cells

Confluent fibroblasts, collected by treatment with 0.05% trypsin-EDTA (Sigma-Aldrich, St. Louis, MO, USA), were subjected to sorting to isolate MUSE cells, as previously described [22,23]. In brief, cells were suspended in an FACS buffer containing 0.5% BSA (bovine serum albumin) and 2 mM EDTA in Fluoro-Brite DMEM (Thermo Fisher, Waltham, MS, USA) and incubated with the anti-mouse SSEA3 antibody (IBL, Fujioka, Japan) for one hour on ice. Subsequently, the cells were washed 3 times with FACS buffer, centrifuged at 400× *g* for 5 min, incubated with an anti-rabbit IgM–FITC antibody (ImmunoReagents, Raleigh, NC USA) for one hour on ice, and again washed 3 times afterward. Magnetic-activated cell sorting (MACS-Miltenyi Biotec, Bergisch Gladbach, Germany) was employed to isolate the SSEA3(+) cells and SSEA3(−) non-Muse cells according to the manufacturer’s instructions (Miltenyi Biotec, Bergisch Gladbach, Germany). The cells were then treated with anti-FITC microbeads (Miltenyi Biotec) for 15 min on ice and then washed with FACS buffer. Finally, the cells were placed on LS columns (Miltenyi Biotec, Bergisch Gladbach, Germany) for magnetic separation.

The collected SSEA3(+) positive cells were cultivated in a suspension on Petri dishes coated with poly 2-hydroxyethyl methacrylate (pHEMA) (Sigma-Aldrich, St. Louis, MO, USA) in low-glucose DMEM containing 10% ES-FBS, 4 mM L-glutamine, 100 U/mL penicillin-streptomycin (HiMedia, Einhausen, Germany), and 2.6% MethoCult (STEMCELL Technologies, Vancouver, BC, Canada) at 37 °C and in 5% CO_2_ for 10 d. Cells were then seeded for subsequent experiments or subjected to analysis.

### 2.4. Neuroglial Differentiation

Muse stem cells (1 × 10^5^) were plated on pHEMA-coated dishes and grown in a neural basal medium containing B27 supplement (Gibco, Waltham, MS, USA), 2 mM L-glutamine, 30 ng/mL bFGF (Peprotech, Cheshire, UK), and 30 ng/mL EGF (Peprotech). After 7 d, cells were dissociated with yellow tips into single cells and plated on 0.1% (wt/vol) dishes coated with poly-L-lysine containing alpha-MEM (MicroGEM) 2% ES-FBS, 25 ng/mL bFGF, and 25 ng/mL BDNF (Sigma). Cells were grown for 2 weeks before analysis.

### 2.5. Full Mature Astrocyte Differentiation

We induced astrocyte differentiation as per the method of Soubannier and colleagues, with some modifications [24]. In brief, for neural induction (1st step), Muse stem cells (1 × 10^5^) were plated on Matrigel-coated dishes and grown in DMEM–F12 (MicroGEM), 1% non-essential amino acids (Sigma), 0.1% bovine serum albumin (Sigma), 2 μM SB431542 reagent (Merck Millipore, Burlington, MS, USA), 200 ng/mL Noggin (PeproTech), 1 mM Glutamax (Life Technologies, Carlsbad, CA, USA), and 1 µg/mL laminin (Sigma). Cells were cultivated until they reached confluence (i.e., 7 d in vitro), after which (2nd step) they were incubated for another 5 d in the same medium without Noggin or SB431542. The medium was changed every day. After this period, the cells were treated with Accutase (Life Technologies) to obtain a single-cell suspension and were transferred to low-binding culture dishes (Corning, Corning, NY, USA). They were then cultivated for 3 d with a neural progenitor medium containing DMEM-F12, 1% non-essential amino acids, 1 mM Glutamax, 1 µg/mL laminin, 20 ng/mL FGF-2, 20 ng/mL EGF, 1% N2, 2% B27, and 1 µM Y-27632 (Sigma). The obtained neurospheres were plated in T25 flasks with astrocyte growth medium (Sigma) and its associated supplement for 30 d. Every 3 d, half of the medium was replaced with fresh medium.

### 2.6. Flow Cytometry Analysis

The cells were washed with PBS and then incubated with anti-CD105, anti-CD90, anti-CD73, anti-CD45, or anti-CD44 PE-conjugated antibodies (Elabscience, Houston, TX, USA). We followed the manufacturer’s instructions for the use of the antibodies. The incubation with antibodies was performed in the dark at room temperature for 30 min. Subsequently, cells were washed with PBS and resuspended in an FACS buffer for analysis on a Guava easyCyte flow cytometer (Merck Millipore, USA). The data analysis was performed with easyCyte software, using the standard procedure. We analyzed at least 5000 cells per sample. Cells were gated for forward-scatter versus side-scatter channel signals. 

### 2.7. Cell Cycle Analysis

For every analysis, 5 × 10^4^ cells were collected and fixed in cold 70% ethanol overnight at −20 °C. The cell samples were washed with PBS and placed in a hypotonic buffer containing propidium iodide (Sigma). The samples were analyzed with Guava easyCyte flow cytometer (Merck Millipore) using easyCyte software and the standard procedure.

### 2.8. Apoptosis Detection by Annexin V Assay

Apoptotic cells were identified by using a fluorescein-conjugated annexin V kit (Dojindo Molecular Technologies, Munich, Germany) on a Guava easyCyte flow cytometer (Merck Millipore). For this procedure, we followed the manufacturer’s instructions. In brief, 5 × 10^4^ cells from each of the different experimental groups were collected and stained with an annexin V-FITC solution containing 7-AAD. The kit contains annexin V and 7-AAD dyes to detect apoptotic and non-apoptotic cells. The green Annexin V binds to phosphatidylserine on the external membrane of apoptotic cells, while the red 7-AAD labels the DNA of late-stage apoptotic and dead cells. This procedure allows for the identification of four different cell populations: Annexin V (−)/7-AAD (−) non-apoptotic cells; Annexin V(+)/7-AAD(−) early apoptotic cells; Annexin V (+)/7-AAD (+) late-stage apoptotic or dead cells; Annexin (V−)/7-AAD (+) necrotic cells. Early-stage and late-stage apoptotic cells were grouped together in our experimental conditions.

### 2.9. Senescence Detection by Acid Beta-Galactosidase Assay

The Muse cell cultures were dissociated into single cells and fixed with 0.2% glutaraldehyde and 2% formaldehyde for 5 min at room temperature. Subsequently, samples were treated with 1 mg/mL X-Gal (GoldBio, St. Louis, MO, USA) staining solution at 37 °C overnight. The percentage of senescent cells was determined according to the number of blue beta-galactosidase-positive cells out of at least 500/600 cells in several microscope fields, as previously reported [25].

### 2.10. Immunocytochemistry

Cells were grown on cover slides and then fixed with 4% formaldehyde for 15 min at room temperature. For the evaluation of stemness, we used the following primary antibodies: SSEA3(IBL), SOX2 (Elabscience, Houston, TX, USA), OCT3/4 (Elabscience) and NANOG (Cell Signaling, Danvers, MS, USA). The Neural Stem Cell Marker Characterization Kit (Millipore, Italy), which contains primary antibodies for NESTIN, SOX2, MAP2, O1, and GFAP, allowed for the identification of neural stem cells, early progenitors, and differentiated cells. The reactions were performed following the manufacturer’s instructions and other published protocols [26]. The identification of astrocytes was also performed using primary antibodies against S100B, EAAT1, EAAT2, APOE, and SOX9 (Elabscience), according to the manufacturer’s instructions. The FITC and TRITC secondary antibodies were obtained from ImmunoReagents. We performed nuclear staining with a DAPI mounting medium (Abcam, Cambrigdge, UK). The micrographs were taken with a fluorescence microscope (Leica, Wetzlar, Germany). The percentage of positive cells was calculated by counting at least 500/600 cells in several microscope fields.

### 2.11. RT-qPCR

We used the RNeasy Mini Kit (Qiagen, Hilden, Germany) to extract the total RNA, following the manufacturer’s instructions. Primers for real-time (RT) PCR reactions were designed with Primer Express (Applied Biosystems, Waltham, MS, USA) using mRNA sequences retrieved from the Nucleotide Data Bank (National Center for Biotechnology Information, Bethessa, MA, USA). The RT-PCR assays were performed using a quantitative PCR machine (Hangzhou Bioer Technology, Hangzhou, China). We performed reactions with SYBR green PCR master mix (ABM, Richmond, BC, Canada) and followed the manufacturer’s instructions. The 2^−ΔΔCT^ method was used for the quantitative RT-PCR data analysis. The primers for the detection of stemness, lineage specification, and differentiation markers were validated by determining the markers’ expression levels in mRNA isolated from human ATCC fibroblasts and from Human Brain Total RNA (Thermo Fisher, Waltham, MS, USA) (Supplementary File S3).

### 2.12. Statistical Analysis

We performed all the above-described experiments in triplicate. We performed a one-way ANOVA and post hoc tests using JASP, an open-source statistics software supported by the University of Amsterdam (https://jasp-stats.org, access date: 5 November 2022).

## 3. Results

According to a procedure already described [20], the peripheral blood and fibroblasts from patients with ID and clinical symptoms overlapping those described for RTT were collected. Mutations in the *IQSEC2* gene may occur in different segments of a coding region and may affect the various corresponding protein domains. We focused our attention on three patients whose mutations modified the C-terminal region of the protein.

Patient 1 showed a deletion at 3260/19 c.3613_3613 delC (p.[Leu1205Trpfs*192]) in the *IQSEC2* gene. The mutation produced a frameshift beginning with codon leucine 1205, altered that amino acid to tryptophan, and caused a premature stop codon at position 192 of the novel reading frame. That mutation disrupted the C-terminus of the IQSEC2 protein (see Supplementary Figure S1). In contrast, Patient 2 had a mutation at 3261/19 c.4110_4111del (p.[Tyr1371Glnfs*15]), and Patient 3 had a duplication at 3259/19 4039 c.4039dup (p.[Ala1347Glyfs*40]). In Patients 2 and 3, the mutations also caused a frameshift with the production of aberrant proteins [20], as shown in Supplementary Figure S1.

All analyses described in the following were performed on Patient 1 and later repeated on the other two patients. Hereinafter, Muse stem cells obtained from Patients 1, 2, and 3 were labeled “Muse-PT1”, “Muse-PT2”, and “Muse-PT3”, respectively. The Muse stem cells isolated from the healthy control were labeled “Muse-CT”. As a further control, we used normal human dermal fibroblasts (HDF) obtained from ATCC and from Lonza Biosciences. The properties of Muse cells isolated from fibroblasts obtained from the control patient and from HDF were similar, and the minimal differences were not statistically significant (data not shown). We then used only the above-indicated Muse-CT as a reference.

### 3.1. Muse Cells Isolated from Fibroblasts of Two Patients with an IQSEC2 Mutation Showed Altered Biological Properties

**Figure 1:**
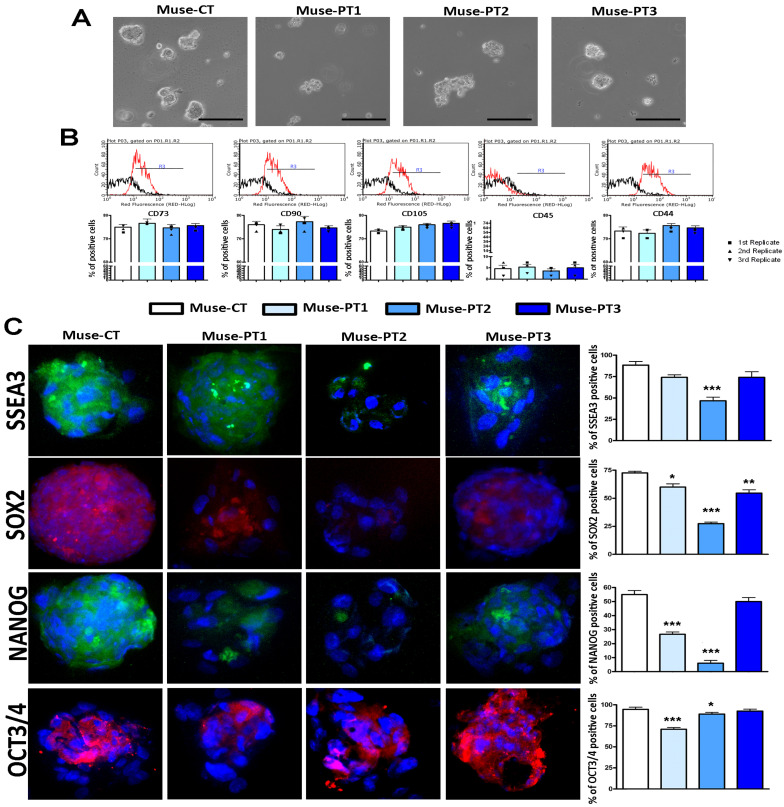
**Markers of Muse stem cells isolated from patients’ fibroblasts**. Panel (**A**): Micrographs showing the typical morphology of Muse stem cells grown in suspension. The black bar corresponds to 100 µm. Panel (**B**): Representative flow cytometry plots of CD73, CD90, CD105, CD45, and CD44 on Muse cells isolated from healthy donors and patients. The histograms indicate the percentage of positive cells for every analyzed marker. Panel (**C**): Representative images of immunocytochemistry analysis to detect SSEA3 (i.e., green), SOX2 (i.e., red), NANOG (i.e., green), and OCT3/4 (i.e., red) in Muse stem cells (200× magnification). Cell nuclei were identified with DAPI (i.e., blue). The graphs on the right indicate the percentages of positive cells in the different samples. For all experiments, the symbols *** (i.e., *p* < 0.001), ** (i.e., *p* < 0.01), and * (i.e., *p* < 0.05) indicate statistical significance between the healthy control and the patients’ samples. For every sample, three biological replicates were performed, and data are reported with SDs.

**Figure 2:**
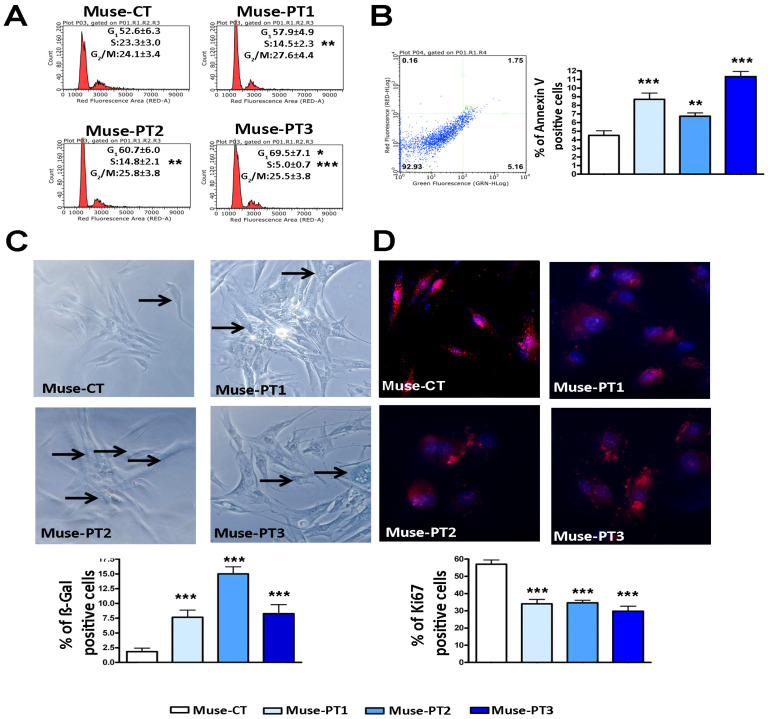
**Biological parameters of patients’ Muse cells.** Panel (**A**): Representative plots of cell cycle analysis on Muse stem cells. Panel (**B**): Example of annexin assay performed on Muse stem cells. The percentage of apoptotic cells in the different samples is shown in the graph. Panels (**C**,**D**): Representative images of beta-galactosidase assay (**C**) and Ki67 immunostaining (**D**) performed on Muse stem cells (200× magnification). The immunostaining identified the Ki67 positive cells, shown in red, while the nuclei were stained with DAPI in blue. In Panel (**C**), some senescent cells in blue are indicated with an arrow. The graphs indicate the percentages of (beta-gal+) senescent cells and (Ki67+) cycling cells, respectively. For all of the experiments, the symbols *** (i.e., *p* < 0.001), ** (i.e., *p* < 0.01), and * (i.e., *p* < 0.05) indicate statistical significance between the healthy control and the patients’ samples. For every sample, three biological replicates were performed, and data are reported with SDs.

The Muse cells isolated from the three patients were SSEA3(+), as illustrated in Figure 1C, and showed other typical markers of stromal cells, including CD44, CD73, CD90, and CD105 (see Figure 1A,B). They were also CD45(−), a general hematopoietic marker [27] (see Figure 1A,B). Nearly all of the cells were OCT3/4 (+) in the Muse-CT, whereas fewer cells expressed the SOX2 and NANOG stemness markers (see Figure 1C). Those results agree with the hypothesis that even in homogeneous stem cell populations, stochastic-state transitions produce a phenotypic equilibrium among different subpopulations possessing different degrees of stemness and differentiation potential [27]. Of note, in Muse-CT, we also observed cytoplasmic staining for SOX2, NANOG, and OCT3/4. It is well known that in embryonic stem cells, these transcription factors may be localized in the cytoplasm, depending on their post-translational status [28,29,30]. The absence of any non-specific staining was demonstrated with a negative immunostaining control (Supplementary File S2). Muse-PT1 and Muse-PT2 showed a reduction in the percentage of NANOG (+) cells (see Figure 1), and Muse-PT2 also showed a decline in the number SOX2 (+) and SSEA3(+) cells. Meanwhile, Muse-PT3 showed a minimal decrease in the percentage of SSEA3(+) cells and a significant decrease in SOX2 cells. The cell cycle profiles of Muse-PT1, Muse-PT2, and Muse-PT3 all showed a reduction in S-phase cells compared with control cultures (see Figure 2A). These results align with the reduction in the percentage of cycling (Ki67+) cells (see Figure 2D). In Muse-PT1, Muse-PT2, and Muse-PT3 cultures, we detected a significant increase in the number of apoptotic cells (see Figure 2B). At the same time, all the patients’ samples showed an increase in the number of senescent cells (see Figure 2C).

### 3.2. Neuroglial Differentiation of Muse Cells

**Figure 3:**
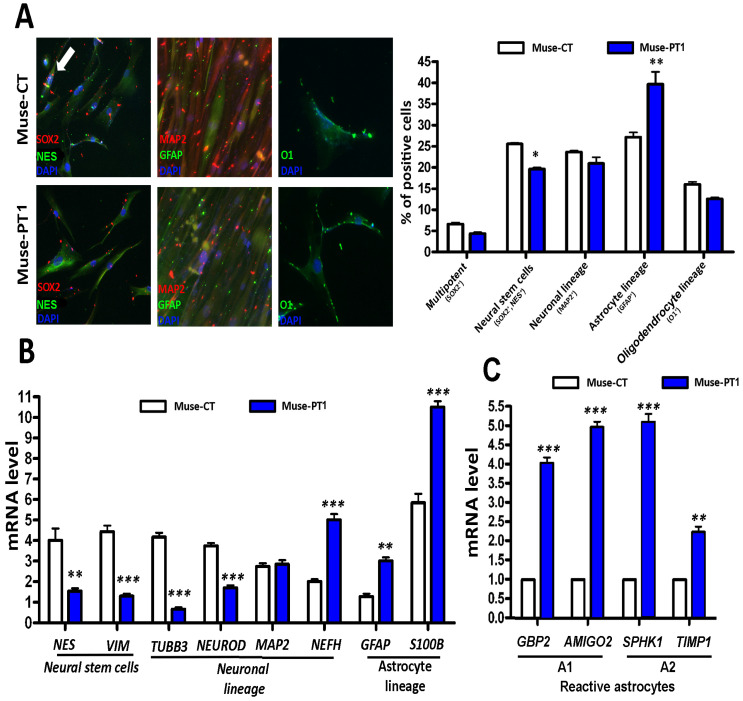
**Neuroglial differentiation of Muse stem cells.** Panel (**A**): Graph showing the percentages of multipotent Muse stem cells (SOX2+), neural stem cells (i.e., SOX2+ and NESTIN+), neuronal (MAP2+), astrocytes (GFAP+), and oligodendrocyte-committed progenitors (O1+) following neuroglial differentiation of Muse stem cells obtained from a healthy control and Patient 1. The micrographs show representative images of immunostaining on differentiated samples (200× magnification). Green and red dots in the background are due to poly-L-lysine coating present in the samples. In Muse-CT sample, the white arrow indicates SOX2/NESTIN-positive cells. Panel (**B**): Histogram showing the mRNA expression level of the indicated genes. The mRNA levels were normalized to GAPDH mRNA expression, which was selected as an internal control. NES = NESTIN; VIM = VIMENTIN. Panel (**C**): mRNA levels of reactive astrocyte markers identified by qRT-PCR. The expression level of markers was set as 1 in the sample obtained from a healthy control (i.e., Muse-CT). For all of the experiments, the symbols *** (i.e., *p* < 0.001), ** (i.e., *p* < 0.01), and * (i.e., *p* < 0.05) indicate statistical significance between the control and Patient 1 (*n* = 3 biological replicates), and data with SD are reported.

We promoted the neural differentiation of Muse cells with a multistep procedure involving the commitment of Muse stem cells to neural stem/progenitor cells (i.e., SOX2+ and NESTIN+), followed by lineage specification in neurons (MAP2+), oligodendrocytes (O1+), and astrocytes (GFAP+). In Muse-PT1 cultures, we detected a decrease in the percentage of neural stem cells and an increase in the number of astrocyte-committed cells (see Figure 3A, Figure S2 for negative immunostaining control). Those data were confirmed in a qRT-PCR analysis of differentiation markers. We observed a decline in the expression of neural stem and progenitor markers (i.e., NESTIN and VIMENTIN) and of immature neuronal lineage markers (i.e., TUBULIN BETA 3 CHAIN and NEUROD1) in each patient’s sample. At odds with that observation, we detected an increase in the neurofilament heavy chain subunit. Other published findings, however, show that aberrant neurofilament expression may be associated with several neurological diseases [31,32].

We also detected a significant increase in the expression of GFAP and S100B, which are typical astrocyte markers (see Figure 3B). However, no changes were detected in OLIG1 mRNA, an oligodendrocyte marker. Following brain injury, astrocytes can undergo a phenotypic transformation into reactive astrocytes, which can either aid the recovery of brain function or further exacerbate pathological conditions; the latter occurs if reactive astrogliosis is dysregulated [33,34]. Reactive astrocytes are generally divided into A1 (i.e., pro-inflammatory) and A2 (i.e., ischemic–anti-inflammatory) astrocytes. In a disease condition, different A1-to-A2 ratios may either promote or impair healing [35]. Astrocyte cultures contain a minimal percentage of reactive cells whose increase can be achieved by mimicking pathological conditions with chemical cues that may specifically induce A1 or A2 astrocytes [36,37]. In the absence of any external cue, the astrocytes that we obtained from Muse-PT1 cells showed a significant increase in the expression of reactive astrocyte markers compared with the control culture (see Figure 3C). In particular, we detected the augmentation of A1 (i.e., GBP2 and AMIGO2) and A2 (i.e., SPHK1 and TIMP1) markers, as shown in Figure 3C [37]. These results suggest that the Muse cells obtained from Patient 1 underwent a dysregulated astrocyte differentiation. Similar results were obtained with samples obtained from Patients 2 and 3 (data not shown).

**Figure 4:**
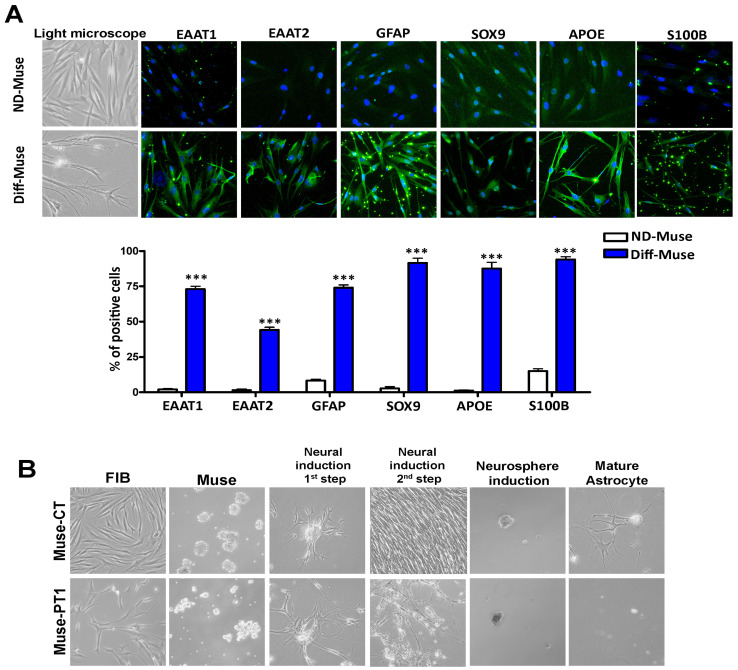
**Astrocyte differentiation of Muse stem cells.** Panel (**A**): Examples of immunostaining on Muse stem cells following astrocyte differentiation (200× magnification). The staining for several astrocyte markers was evaluated in non-differentiated and differentiated Muse stem cells (ND-Muse; Diff-Muse). Some green background dots are due to Matrigel present on the samples. The cells’ appearance under light microscopy is also shown. The histogram reports the percentages of cells expressing the analyzed markers. For all experiments, the symbol *** (*p* < 0.001) indicates statistical significance between the differentiated and non-differentiated cells (*n* = 3 biological replicates), and data with SD are reported. Panel (**B**): Phase contrast microscope images of fibroblasts (FIB) isolated from a healthy control and Patient 1 (200× magnification). The figure also shows the undifferentiated Muse cells (Muse) isolated from the corresponding fibroblast cultures and the Muse cells induced to become astrocytes through a four-step procedure: the 1st and 2nd steps of neural induction, neurosphere induction, and mature astrocytes. The patient’s samples failed to become mature astrocytes, and the impairment of differentiation process was already evident at neural induction 2nd step.

We decided to further investigate the process of astrocyte differentiation in cells with the mutated IQSEC2 gene and performed an in vitro procedure to obtain fully mature astrocytes [24,38]. The differentiation protocol preliminarily involved the production of neural stem cells growing in suspension as neurospheres; these stem/progenitor cells can be induced to differentiate into mature astrocytes. The Muse stem cells from the healthy control produced mature astrocytes which expressed several markers typically present in differentiated astrocytes [24], as shown in Figure 4A (see Figure S2 for the negative immunostaining control).

The Muse-PT1, Muse-PT2, and Muse-PT3 cells reached the neurosphere stage, but failed to become mature astrocytes because the treatment with the glial differentiation medium induced cell death (Figure 4B). This result further evidenced that in patients with IQSEC2-related diseases, the neural stem and progenitor cells showed a dysregulation of lineage specification that may produce a high percentage of cells committed to astrocyte lineage (see Figure 3) but not fully differentiated astrocytes. Indeed, the committed cells died when the differentiation procedure was performed to obtain mature astrocytes.

## 4. Discussion

Stem cells have been used as models for human pathologies. The vast majority of adult stem cells, including hematopoietic and adipose-derived stem cells, may be useful to study tissue-specific diseases but cannot be used as general models for investigating human illnesses, given their limited differentiation potential compared with iPSCs. The discovery of Muse stem cells, which possess multilineage potential, may represent a physiological alternative to iPSCs that are obtained through an in vitro procedure of reprogramming somatic cells.

Against that background, we demonstrated that the population of Muse cells present in fibroblast cultures obtained from biopsies of patients’ skin may be induced to differentiate into cells of interest for analyzing diseases. We evaluated the biological properties of Muse cells from three patients with ID and with mutations in the *IQSEC2* gene. The patients’ cultures showed a reduced number of cycling cells and increased senescence compared with the control cultures. Cells obtained from two of the patients also showed an augmented rate of apoptosis. Those results indicate that the impairment of IQSEC2 protein functions may affect the biological properties of stem cells.

The neuroglial differentiation of Muse cells further demonstrated that *IQSEC2* mutations may alter the self-renewal and lineage specification of stem cells. In particular, we detected a decrease in the percentage of neural stem/progenitor cells (i.e., SOX2+ and NESTIN+) in cultures obtained from Muse cells with the mutated *IQSEC2* gene. This alteration in the number of stem and progenitor cells produced a bias toward a commitment to astrocyte lineage and early steps of differentiation. The concurrent expression of reactive astrocyte markers (i.e., A1 pro- and A2 anti-inflammatory phenotypes) in the absence of any inducing cue indicates that astrogenesis differentiation may produce flawed instead of functional astrocytes. This hypothesis is strengthened by the observation that the differentiation protocol to obtain mature astrocytes failed to produce fully differentiated cells from the patients’ cultures.

The *IQSEC2* gene may harbor mutations that affect different domains of the corresponding proteins, which may contribute to a high variability in clinical outcomes. We evaluated cells obtained from three patients with mutations that produce modifications in the C-terminal region of IQSEC2 proteins where the PDZ domain in located. This domain is found in scaffolding proteins, which regulate post-synaptic receptor and signaling proteins [19]. PDZ domains are protein–protein interaction modules found in many proteins; thus far, 928 PDZ domains have been identified in 328 proteins, which are present either in single or multiple copies or in combination with other interaction modules [39]. The mutation in the PDZ domain of IQSEC2 may affect ARF6 activation and consequently attenuate a large number of downstream signaling pathways including YAP/TAZ, which plays a key role in the lineage commitment of neural stem cells [40]. The PDZ-RSG3 protein has also been shown to be involved in the maintenance of neural progenitor cells [41]. These findings suggest that the alteration of neuroglial commitment and differentiation we detected may relate to the alteration of PDZ signaling. Moreover, the mutations affecting the PDZ domain may also be related to “feckless” astrocytes with altered neurotransmitter transporters [42].

It remains to be determined whether *IQSEC2* mutations affecting other protein domains may determine a similar modification of neural commitment and differentiation with a bias toward dysregulated astrogenesis. At the same time, whether other mutations modifying the C-terminal region of *IQSEC2* may produce the same biological outputs that we detected should be evaluated.

## 5. Conclusions

Our research has demonstrated that Muse stem cells, which are physiological multipotent stem cells present in the body, may be useful as disease modeling systems.

## Data Availability

A list of primers for the RT-PCR and other details of the experimental procedure may be provided upon request.

## Supplementary Materials

The following supporting information can be downloaded at: https://www.mdpi.com/article/10.3390/cells12070977/s1. Figure S1: IQSEC2 mutations in analyzed patients with ID. Figure S2: Negative controls of immunostaining experiments. Figure S3: Validation of the primers for detection of stemness, lineage specification, and differentiation markers.
