# Supplementary material for: An Example of Neuro-Glial Commitment and Differentiation of Muse Stem Cells Obtained from Patients with IQSEC2-Related Neural Disorder: A Possible New Cell-Based Disease Model
Source: Cells. 2023 Mar 23;12(7):977. doi: 10.3390/cells12070977 (PMC10093355; doi:10.3390/cells12070977)
Supplement: Supplementary file 1 [file cells-12-00977-s001.zip › Sup files/Figure S1.pdf]

Homo sapiens IQ motif and Sec7 domain ArfGEF 2 (IQSEC2), transcript variant 1, mRNA

NCBI Reference Sequence: NM_001111125.3

WT ggg tgg cgc cgt gag aga gcg ggg aca ggg ctc tcc tgc ccg ttc ccc tgc ccc tgg gcc gcc agg **atg** gag gcg ggg
PT1 ggg tgg cgc cgt gag aga gcg ggg aca ggg ctc tcc tgc ccg ttc ccc tgc ccc tgg gcc gcc agg **atg** gag gcg ggg
PT2 ggg tgg cgc cgt gag aga gcg ggg aca ggg ctc tcc tgc ccg ttc ccc tgc ccc tgg gcc gcc agg **atg** gag gcg ggg
PT3 ggg tgg cgc cgt gag aga gcg ggg aca ggg ctc tcc tgc ccg ttc ccc tgc ccc tgg gcc gcc agg **atg** gag gcg ggg

...

WT ccc ccg ccg cca gag gag tac aag agc cag agg ccc gtc tcc aac tcc tca tcc ttc ctg ggc tcc ctt ggg ctc cct
PT1 ccc ccg ccg cca gag gag tac aag agc cag agg ccc gtc tcc aac tcc tca tcc ttc -tg ggc tcc ctt ggg ctc cct
PT2 ccc ccg ccg cca gag gag tac aag agc cag agg ccc gtc tcc aac tcc tca tcc ttc ctg ggc tcc ctt ggg ctc cct
PT3 ccc ccg ccg cca gag gag tac aag agc cag agg ccc gtc tcc aac tcc tca tcc ttc ctg ggc tcc ctt ggg ctc cct

...

WT ggc cca gtc cca ggg ccc caa cac tat acc ttg ggc cgg cca ggc agg gca ccc aga cgg ggg gct gga gga
PT1 ggc cca gtc cca ggg ccc caa cac tat acc ttg ggc cgg cca ggc agg gca ccc aga cgg ggg gct gga gga
PT2 ggc cca gtc cca ggg ccc caa cac tat acc ttg ggc cgg cca ggc agg gca ccc aga cgg ggg gct gga gga
PT3 ggc cca gtc cca ggg ccc caa cac tat acc ttg ggc cgg cca ggc agg gca ccc aga cgg ggg **G**gct gga gga

WT cac cct cag ttt gct cca cat ggc cgc cac ccc ctg cac cag ccc aca tcc cca ctg ccc ctg tac agt cct gcc
PT1 cac cct cag ttt gct cca cat ggc cgc cac ccc ctg cac cag ccc aca tcc cca ctg ccc ctg tac agt cct gcc
PT2 cac cct cag ttt gct cca cat ggc cgc cac ccc ctg cac cag ccc aca tcc cca ctg ccc ctg ta- agt cct gcc
PT3 cac cct cag ttt gct cca cat ggc cgc cac ccc ctg cac cag ccc aca tcc cca ctg ccc ctg tac agt cct gcc

WT ccc cag cac cct cca gcc cac aaa cag ggc cct aag cac ttc atc ttc agc cac cac cca cag atg atg cca
PT1 ccc cag cac cct cca gcc cac aaa cag ggc cct aag cac ttc atc ttc agc cac cac cca cag atg atg cca
PT2 ccc cag cac cct cca gcc cac aaa cag ggc cct aag cac ttc atc ttc agc cac cac cca cag atg atg cca
PT3 ccc cag cac cct cca gcc cac aaa cag ggc cct aag cac ttc atc ttc agc cac cac cca cag atg atg cca

WT gca gca ggc gcg gct ggg ggc cct gga tcc cgg cca cca ggg ggc tcc tac tcc cac ccc cac cac ccc cag
PT1 gca gca ggc gcg gct ggg ggc cct gga tcc cgg cca cca ggg ggc tcc tac tcc cac ccc cac cac ccc cag
PT2 gca gca ggc gcg gct ggg ggc cct gga tcc cgg cca cca ggg ggc tcc tac tcc cac ccc cac cac ccc cag
PT3 gca gca ggc gcg gct ggg ggc cct gga tcc cgg cca cca ggg ggc tcc tac tcc cac ccc cac cac ccc cag

WT tca cca ttg tca cca cac tca ccc atc cca ccc cac ccc tcc tat cca ccc ctc ccc cca ccc tcc cct cac acc
PT1 tca cca ttg tca cca cac tca ccc atc cca ccc cac ccc tcc tat cca ccc ctc ccc cca ccc tcc cct cac acc
PT2 tca cca ttg tca cca cac tca ccc atc cca ccc cac ccc tcc tat cca ccc ctc ccc cca ccc tcc cct cac acc
PT3 tca cca ttg tca cca cac tca ccc atc cca ccc cac ccc tcc tat cca ccc ctc ccc cca ccc tcc cct cac acc

WT ccg cac tca ccc ctt cca ccc acc tcc ccc cat ggc ccg ctg cac gcc tct ggg ccc cct ggc aca gcc aac
PT1 ccg cac tca ccc ctt cca ccc acc tcc ccc cat ggc ccg ctg cac gcc tct ggg ccc cct ggc aca gcc aac
PT2 ccg cac tca ccc ctt cca ccc acc tcc ccc cat ggc ccg ctg cac gcc tct ggg ccc cct ggc aca gcc aac
PT3 ccg cac tca ccc ctt cca ccc acc tcc ccc cat ggc ccg ctg cac gcc tct ggg ccc cct ggc aca gcc aac

WT ccc ccc agt gca aac ccc aag gcc aag cca agc cgg atc agc acc gtg gtc tga tga atg gag aga gtg agc
PT1 ccc ccc agt gca aac ccc aag gcc aag cca agc cgg atc agc acc gtg gtc tga tga atg gag aga gtg agc
PT2 ccc ccc agt gca aac ccc aag gcc aag cca agc cgg atc agc acc gtg gtc tga tga atg gag aga gtg agc
PT3 ccc ccc agt gca aac ccc aag gcc aag cca agc cgg atc agc acc gtg gtc tga tga atg gag aga gtg agc

Supplementary Figure 1 – IQSEC2 mutations in analyzed patients with ID

The coding region of the IQSEC2 gene (NM_001111125.3) in normal individuals (WT). The green and red boxes denote the ATG start codon and the TGA stop codon, respectively. In Patient 1 (PT1) and Patient 2 (PT2), a nucleotide deletion (i.e., pink oval) produced a frameshift with a premature stop codon (i.e., yellow boxes). In Patient 3 (PT3), a guanine insertion (i.e., pink oval) also generated a premature stop codon (i.e., yellow box).
