# Supplementary figures and images for: An Example of Neuro-Glial Commitment and Differentiation of Muse Stem Cells Obtained from Patients with IQSEC2-Related Neural Disorder: A Possible New Cell-Based Disease Model
Source: Cells. 2023 Mar 23;12(7):977. doi: 10.3390/cells12070977 (PMC10093355; doi:10.3390/cells12070977)

MUSE

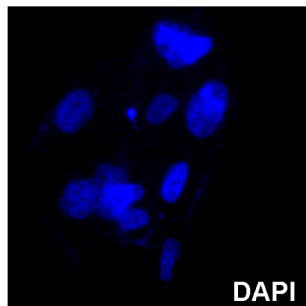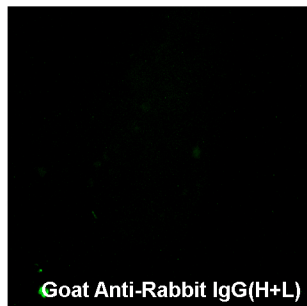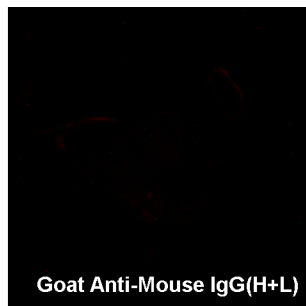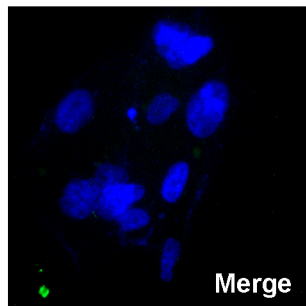

Neural Induction

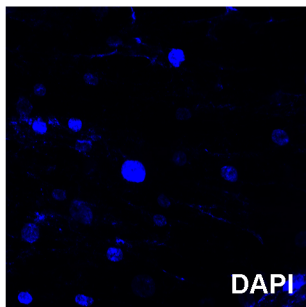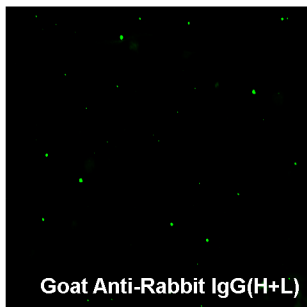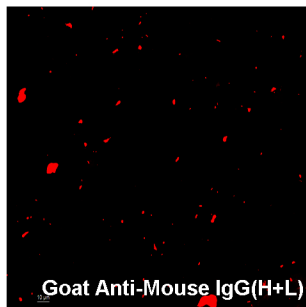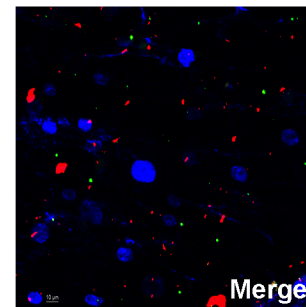

Astrocyte Differentiation

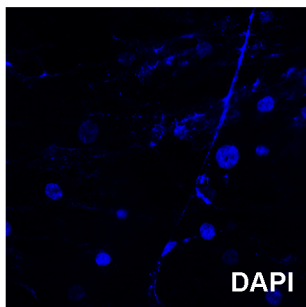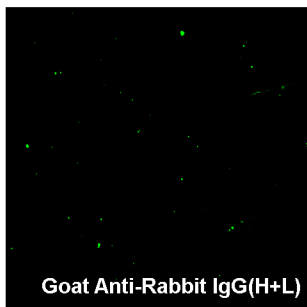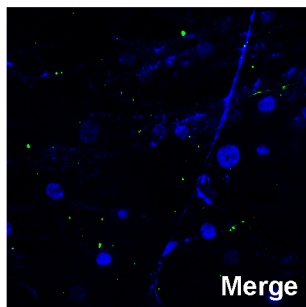

Supplement: Supplementary file 1 [file cells-12-00977-s001.zip › Sup files/Figure S2.pdf]

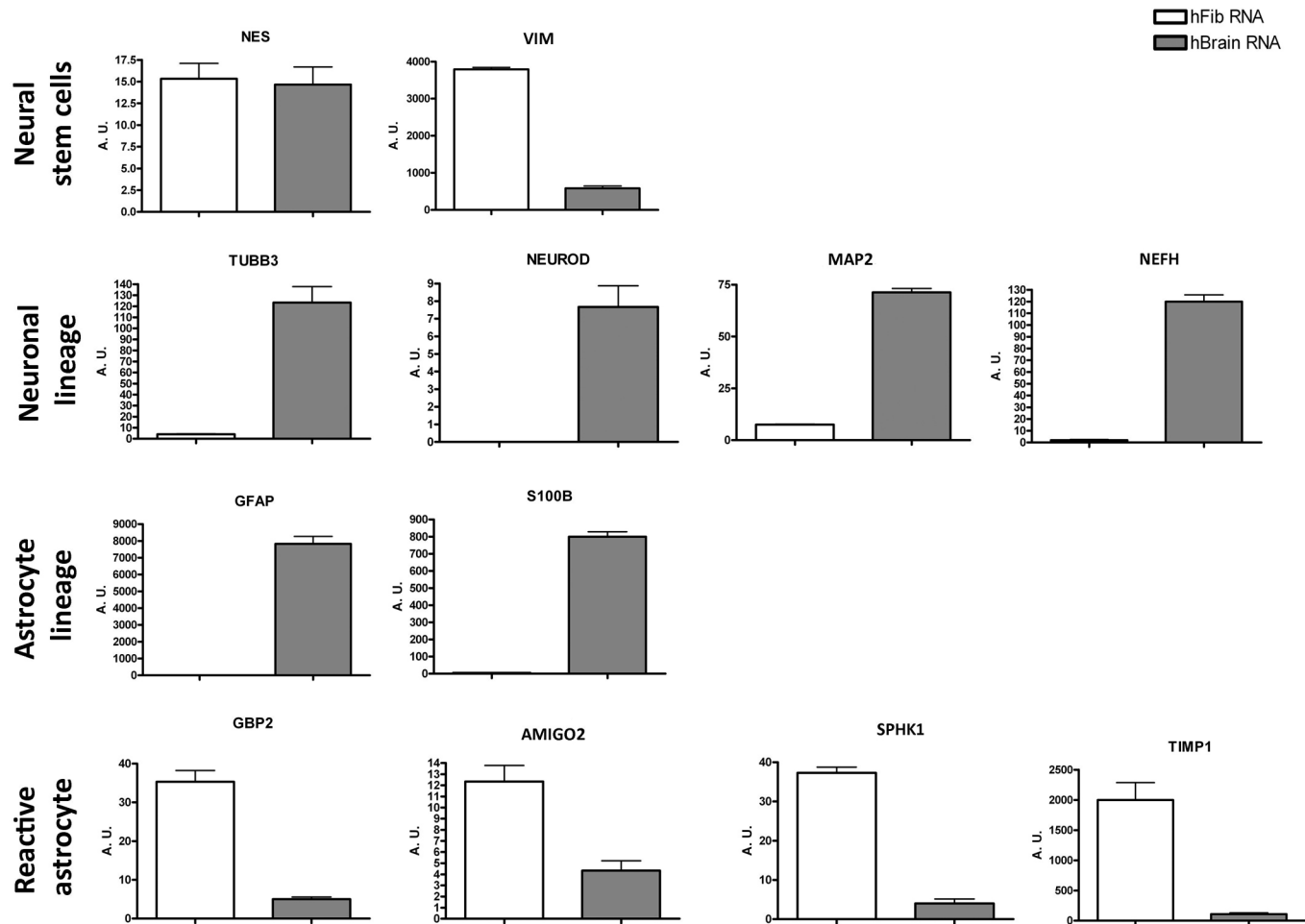

Supplement: Supplementary file 1 [file cells-12-00977-s001.zip › Sup files/Figure S3.pdf]
